# Dramatic portrayal of suicide: a critical analysis of Netflix's 13 Reasons Why

**DOI:** 10.1192/bjo.2021.779

**Published:** 2021-06-18

**Authors:** Anna Sutton, Ross Overshott

**Affiliations:** 1 University of Manchester; 2 Greater Manchester Mental Health NHS Foundation Trust

## Abstract

**Aims:**

The aims of this project were to assess how well the Netflix drama 13 Reasons Why portrayed suicide, in terms of both accuracy and safety, and to discuss the potential effect this could have on viewers.

**Background:**

Psychiatric content within dramatic media can have measurable effects on the population, such as reinforcing stigma around mental illness. Given the show's focus on a character's suicide, the most serious effect here would be suicide contagion.

Guidelines and regulations for the portrayal of suicide in media are in place to protect those who might be vulnerable to suicide contagion.

**Method:**

We formed our own pro-forma of 42 criteria using existing guidelines written for both news and dramatic media. These criteria were formatted into positive and negative pairs; positive being instances of guidelines being followed, negative as guidelines being broken. These were further organised into 7 categories.

Each episode of seasons 1-2 was then assessed against the criteria. Cumulative instances of guidelines being followed or broken were compared within and between seasons. Context of each instance was taken into account by the primary researcher, and we also highlighted instances of exceptional breach of these guidelines.

**Result:**

The results showed an over-all breach of the guidelines, with no significant improvement between the seasons. Some categories of criteria, such as “asking for help” and “mental health”, were portrayed well overall. Other categories, such as “blame”, performed extremely badly.

The most significant breach was the graphic suicide scene at the end of the first season, which completely disregarded Samaritans’ guidelines.

**Conclusion:**

The breaching of guidelines in this show was overwhelming. In terms of severity, although there were some positive themes running through the seasons, there were also worrying instances of guidelines being completely disregarded. This led to the conclusion that the producers of the show did not take their responsibility to young, vulnerable viewers seriously regarding the dangers around portraying suicide.

Suggestions from this study are that more guidelines around suicide are needed specifically for dramatic media, and that existing guidelines should be conflated and have stronger implementation by regulators. This implementation should potentially include overseas providers such as Netflix. Ethically, a significant challenge here is maintaining balance between safety and allowing artistic licence.

